# Unique expression pattern of circulating exosomal miRNA correlates with CNS pathology

**DOI:** 10.3389/fimmu.2026.1848236

**Published:** 2026-07-09

**Authors:** Krzysztof Selmaj, Magda Tinsley, Jerome Badaut, Marcin P. Mycko

**Affiliations:** 1 Department of Neurology and Neurosurgery, University of Warmia and Mazury, Olsztyn, Poland; 2 Center of Neurology, Lodz, Poland; 3 Centre d’Etudes Biologiques de Chizé (CEBC), Villiers-en-Bois, France

**Keywords:** demyelination, exosome, miRNA, multiple sclerosis, traumatic brain injury

## Abstract

**Background and objectives:**

To investigate the total circulating exosome transcriptome in animal models of two different CNS pathologic conditions, autoimmune demyelination and traumatic brain injury (TBI).

**Methods:**

Next generation sequencing (NGS) was used to define the global RNA profile of blood exosomes in the MOG-induced EAE model of autoimmune demyelination and TBI mice model. We analyzed 5 million reads per sample, including a detailed analysis of microRNAs (miRNAs) differentially expressed in EAE versus control mice and versus TBI model.

**Results:**

Exosomal RNA NGS revealed 10 different classes of transcripts including miRNA. MiRNA numbers from EAE and TBI mice showed trend to be lower versus control mice. The differentially expressed miRNAs were found in all three sequential EAE time points: early stage post mice immunization (EAEp), peak of disease (EAEa) and recovery (EAEl). The differential expression pattern of miRNA in EAE versus TBI correlated with their involvement in activation or inhibition of immune response. In the early stage of EAE miRNA-335-5p and miRNA-411-5p were upregulated versus TBI. At the peak of disease six miRNAs were most upregulated, miRNA-27a-3p, miRNA-141-3p, miRNA27b-3p, miRNA-195a-5p, miRNA21a-5p and miRNA-99a-5p. The six most upregulated miRNA at the recovery time point of EAE, miRNA-27b-3p, miRNA 141-3p, miRNA 27a-3p, miRNA-127-3p, miRNA-429-3p and let-7b-5p were associated with inhibition of immune response and enhancement of immunoregulation. Interestingly, it was found that similar pattern of downregulated miRNAs versus TBI were present at all three of EAE time points. Bioinformatic analysis of protein genes targeted by the most differentially expressed miRNAs revealed a numbers of proteins involved in neuroinflammation both in EAE and TBI models. In the early phase of EAE, three protein transcripts, Hdac9, Syt7 and NT5c2 serve as an epigenetic switch controlling T cell fate and cytokine production. In recovery of EAE RoRb, a member of ROR nuclear receptor family involved in Th17 cell differentiation was inhibited.

**Discussion:**

These data indicate that different type of CNS pathology, autoimmune demyelination versus TBI is associated with different pattern of miRNA expression in circulating exosomes which may suggest their differential role in these two CNS pathologies.

## Introduction

The epigenetic regulation may significantly impact different type of pathologies. MicroRNA (miRNA) represent a critical epigenetic mechanism controlling cell transcriptional activity. Differential expression and activation of miRNA may importantly modulate development of pathologic processes (1). In the CNS neuroinflammation represent a uniform reaction to different types of harmful events (2). Classically neuroinflammation was associated with inflammatory and immune mediated disorders. However, more recently neuroinflammation flare was described in several other conditions, stroke, neurodegeneration, traumatic brain injury. The exact role of epigenetic mechanism operating in different types of neuroinflammation is not known. MiRNAs are endogenous 22nt noncoding RNAs that post-transcriptionally regulate gene expression and function. They provide a guide for proteins involved in the function of RNA-induced silencing complexes that degrade complimentary target mRNAs or block their translation (3). Thus, miRNA represent an important regulatory mechanism significantly involved in transcription regulation. Accordingly, miRNA have been shown to heavily influence several biological processes including brain disorders (4).

Exosomes are microvesicles of a size between 30 and 100nm, which serve as small membranous vesicles that are formed in the cytoplasm and released from the surface of almost all living cells. They are known to contain a number of compounds, including proteins, lipids, mRNA, and miRNA, as well as double-stranded DNA (5). Exosomes are also believed to provide a means of cell-to-cell communication by transporting their cargo and delivering it to target cells. Exosomes have been shown to be picked up by other cells and to transfer their contents intracellularly, which can influence the function of the recipient cell (6). An important component of exosome cargo involve non-coding RNA species like microRNA (miRNA).

In this study we compare the exosome miRNA content in two different pathologic conditions of brain injury, demyelinating autoimmune encepaholmyelities and traumatic brain injury (TBI). We addressed the question how specific is exosome miRNA content in relation to different mechanisms of brain damage and how exosome miRNA might contribute to the underlying pathology. We have found strikingly different quantitative and qualitative expression pattern of miRNA in circulating exosomes from EAE mice versus mice with TBI suggesting diverse epigenetic pathways controlling both conditions.

## Methods

### Experimental autoimmune encephalitis

EAE model induced with MOG peptide in C57Bl/6 mice have been used in this study (7). Eight- to 12-week-old mice were immunized subcutaneously over the abdominal flanks with 0.15 mg of MOG peptide in 150ul of CFA containing 0.75 mg of Mycobacterium tuberculosis (Difco Laboratories). In addition, 0.2 g of pertussis toxin (Sigma) was injected into a tail vein on days 0 and 2. Mice were observed daily up to day 60 for neurologic signs of EAE and were scored on the scale from 0 to 5 as follows: 0 no disease; 1 weak tail or wobbly walk; 2 hindlimb paresis; 3 hindlimb paralysis; 4 hindlimb and forelimb paralysis; and 5 death.

### Traumatic brain injury models

Closed head injury model with focal injury, head acceleration, and rotational component was performed as previously described (8). Briefly, C57Bl/6 mice were anaesthetized using 2.5% isoflurane and 1.5 l/min air for 5 min and impacted directly over their intact head using an electromagnetic impactor (Leica Impact One Stereotaxic impactor, Leica Biosystems, Richmond, IL, USA) with a 3 mm round tip at a speed of 3 m/s, a depth of 3 mm and a dwell time of 100 ms. The tip of the impactor was placed over the left somatosensory-parietal cortex center at ~ Bregma 1.7 mm. Sham mice underwent the same anesthesia procedure and were placed under the impactor, but did not receive any impact. Mice were allowed to fully recover in an individual cage before being reintroduced to their home cage.

### Isolation of exosomes from the blood

Exosomes were isolated by the polymer formulation method (9) from blood samples (250ul) using an ExoQuick kit (System Biosciences, Mountain View, CA) according to the manufacturer’s protocol. Isolated exosomes were processed for total exosomal RNA extraction using SeraMir Exosome RNA Amplification kit (System Biosciences) according to the manufacturer’s protocol. After isolation, RNA purity and quantification analysis was performed by an RNA 6000 Pico kit and Small RNA kit (Agilent Technologies, Santa Clara, CA) on the Agilent Bioanalyzer 2100 system.

### Isolation and characterization of exosomes from EAE and TBI mice

**Figure 1:**
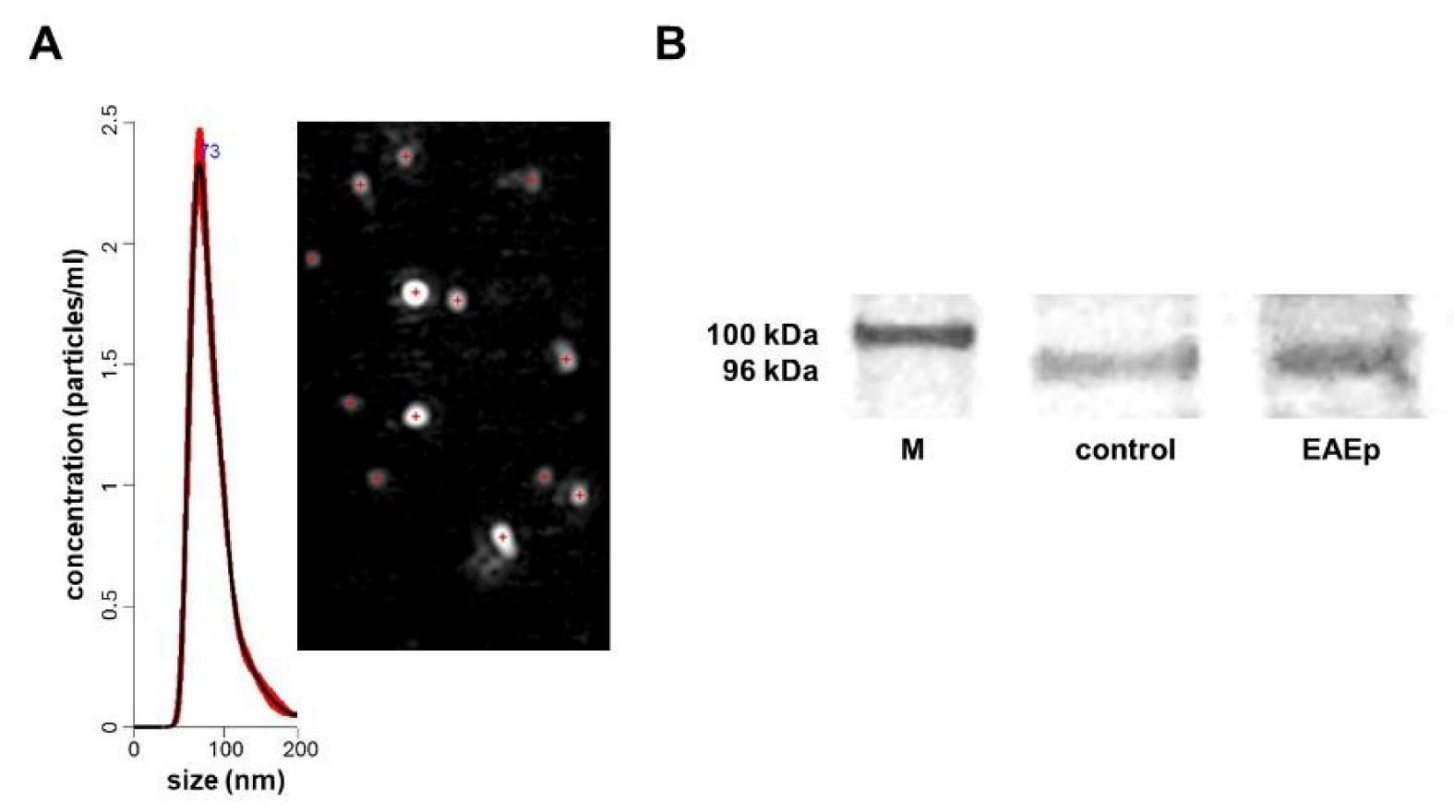
Analysis of circulating exosomes. **(A)** Example of nanoparticle tracking analysis (NTA) video frame and NTA measurements of exosome size in the blood. **(B)** representative serum exosomal sample Western blot analysis of marker proteins PDCD6IP/Alix 96kDa. (M) protein weight marker, control and EAEp.

Exosomes were isolated from mice blood of two experimental models, EAE and TBI. For EAE there was 4–5 mice per each time point group and for TBI 6 to 10 mice. Exosomes were isolated from EAE animals shortly after immunization (EAEp) – day 6, at the peak of disease (EAEa) – day 12 and at the stage of disease recovery (EAEl) – day 40. In TBI model, exosomes were isolated 3 and 6 months post-concussion (TBI3m) and (TBI6m), respectively. Vesicles isolated from the blood were confirmed to be exosomes based on size (30–100nm) as determined by NTA and by expression of exosomal marker protein Alix (**Figures 1A, B**).

### Nanoparticle tracking analysis of exosomes

Nanoparticle tracking analysis (NTA) measurements (10) were performed using a NanoSight NS500 instrument (Malvern Instruments, Malvern, UK). The principle of NTA is based on the technique of direct detection and visualization of individual nanoparticles in suspension, which provides information about particle size, concentration, and distribution. Particles are tracked through light-scattering from a laser source, and the paths are calculated over time to determine their velocity due to Brownian motion. Exosomes were diluted with phosphate- buffered saline (PBS) and disaggregated using a syringe and needle (29 gauge). Samples were then injected into a NanoSight sample cubicle. The mean 6 standard deviation size distribution of exosomes and the mean number of particles per milliliter were determined.

### Western blotting

For Western blot analysis, the circulating exosome pellet isolate using ExoQuick exosome precipitation solution was resuspended in radioimmunoprecipitation assay buffer (25mM Tris-HCl [pH 7.6], 150mM NaCl, 1% NP-40, 1% sodium deoxycholate, 0.1% sodium dodecyl sulfate [SDS]; Sigma- Aldrich, St Louis, MO), with the appropriate protease inhibitor cocktail (Sigma-Aldrich), and then boiled for 5 minutes at 95°C in 2x Laemmli buffer (0.125M Tris-HCl [pH 6.8], 20% glycerol, 4% SDS, 10% 2-mercaptoethanol, 0.004% bromophenol blue). The lysed exosomal proteins were separated by standard SDS–polyacrylamide gel electrophoresis and transferred onto a polyvinylidene fluoride membrane (Millipore, Billerica, MA). The membrane was first incubated in blocking buffer containing 5% nonfat milk, in Tris-buffered saline with Tween (TBST) buffer (20mM Tris-base, 137mM NaCl [pH 7.6], 0.05% Tween 20; Sigma-Aldrich), for 1 hour at room temperature and then probed overnight at 4°C with monoclonal anti-mouse PDCD6IP/Alix antibody (from Affinity Biosciences). After washing 3 times in TBST buffer, blots were incubated for 1 hour at room temperature with an appropriate horseradish peroxidase–conjugated secondary antibody (peroxidase AffiniPureGoat Anti-Mouse IgG H+L from Jackson Immuno Research Europe LTD). An enhanced chemiluminescence Western blotting detection system (ECLplus, PerkinElmer, Boston, MA) was used as the visualizing agent, according to the manufacturer’s protocol.

### Exosomal RNA library preparation and NGS

Small RNA libraries were prepared using the NEXTFLEX^®^ Small RNA-Seq Kit v3 (PerkinElmer, Rodgau, Germany). Briefly, the NEXTflex 3’ and NEXTflex 5’ 4N Adapter were diluted 1:2, and the amplification was performed with 18 cycles. The amplified libraries were purified with AMPure XP beads (Beckman Coulter, Krefeld, Germany) by using a 2:1 bead:sample ratio. The purified libraries were quantified with Fragment Analyzer (AATI) and sequenced on a NextSeq500 system.

### RNA-seq data analysis and differential expression analysis

Mapping steps were performed with bowtie v1.2.2 and miRDeep2 v2.0.1.2 (11), whereas reads were mapped first against the genomic reference GRCm38.p6 provided by Ensembl (12), allowing for two mismatches and subsequently miRBase v22.1, filtered for miRNAs of mmu only, allowing for one mismatch. For a general RNA composition overview, non-miRNA mapped reads were mapped against RNA central and then assigned to various RNA species of interest. Statistical analysis of preprocessed NGS data was done with R v4.0 and the packages pheatmap vNA, pcaMethods v1.82 and genefilter v1.72. Differential expression analysis with edgeR v3.32 (13) used the quasi-likelihood negative binomial generalized log-linear model functions provided by the package. The independent filtering method of DESeq2 (14) was adapted for use with edgeR to remove low abundant miRNAs and thus optimize the false discovery rate (FDR) correction.

### Exosomal miRNA target prediction

The classification of protein-coding mRNA putatively controlled by identified exosomal miRNA was performed with the help of the miRNA target prediction database miRWalk (version 3.0; http://mirwalk.umm.uni-heidelberg.de/about/). miRWalk contains information that applies a random forest-based approach to integrate and predict miRNA target sites. The output of the random forest model is the predicted probability that a candidate target site is a true target site. Each of the miRNAs predicted to be affected by circRNAs is queried into the miRWalk to produce a list of candidate mRNAs with a predicted P-value <0.05.

### Standard protocol approvals, registrations, and patient consents

All of the study procedures have been approved by the University of Warmia and Mazury, Local Ethics Committee for Animal Experiments, permit number 100/2019.

### Statistical analysis

Differentially expressed miRNAs were chosen if significant differences (p < 0.05) among the groups were observed. In comparative analysis between samples of different time points of EAE and TBI groups vs control, a 1-way analysis of variance (ANOVA) test with Scheffe *post hoc* test were used. Prior to the ANOVA test, Levene Test for Equality of Variances was performed. The probability values from each analysis were also adjusted for multiple comparisons using the approach of Benjamini and Hochberg to control for false discovery rates at 0.05. Outlier sensitivity analysis of the serum exosome miRNA concentrations was tested using the Tukey method value (Tukey test).

### Data availability

The data presented in this paper will be available at the authors request.

## Results

### NGS of the exosome RNA content

**Figure 2:**
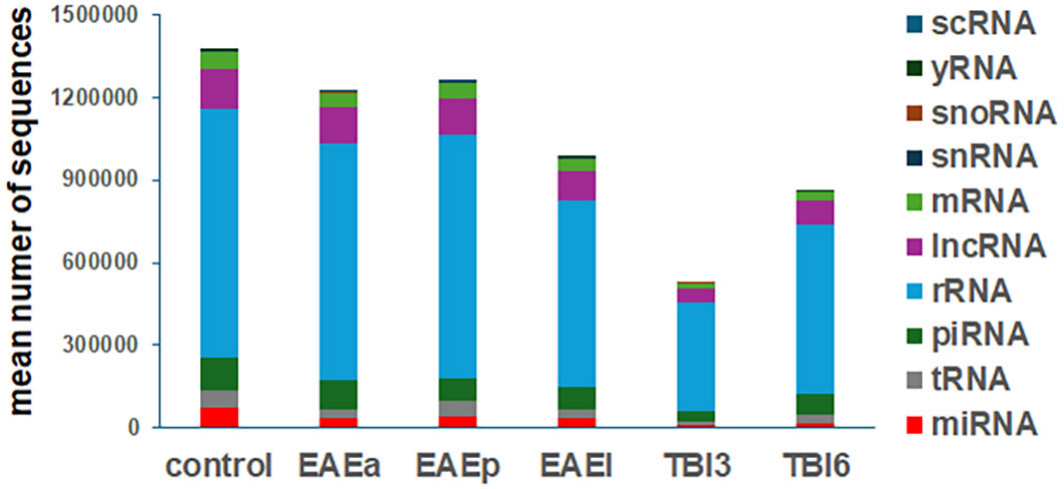
The average mean number of sequences per sample of different classes of RNA detected in circulating exosomes in control, EAE and TBI mice.

To determine a global profile of the exosomal transcriptome from the blood of EAE and TBI mice, we have tested samples from EAE and TBI mice using an RNA-seq. Each sample processed for NGS represents an individual mouse. NGS of these samples yielded on average 5 million reads aligned to the reference mice genome sequence. These reads corresponded to the 222528 miRNA sequences and 652 different miRNA species (**Table 1**). We categorized exosomal RNA into the following 10 different classes: miRNA, tRNA, piRNA, rRNA, lncRNA, mRNA, snRNA, yRNA, scRNA, other RNA. All RNA classes were present in exosomes from EAE and TBI model groups (**Figure 2**). These data confirm that a large portion of the transcriptome is present extracellularly in the circulating exosomes and that miRNA constitute a significant portion of it. The highest number of miRNA sequences was found in exosome from control mice. However, the difference between EAE and control mice was not significant. The number of miRNA specieswere relatively equally distributed across all sample groups (**Table 1**). These data suggest that during pathologic processes, both autoimmune and in particular traumatic, the amount of miRNA in circulating exosomes is decreased in comparison to control animals.

**Table 1:** Number of miRNA sequences and types identified in exosomes from control, EAE and TBI mice.

Model	Group	Nr of miRNA seq	Nr of miRNA types
Control		74922	393
EAE	EAEp	43497	294
	EAEa	34638	339
	EAEl	36561	360
TBI	3m	14414	233
	6m	18494	415
**TOTAL**		**222528**	

EAEp, early EAE; EAEa, peak of disease; EAEl, recovery; 3m, 3 months; 6m, 6 months.

Bold values highlight the number of total sequences.

### Exosomal miRNA expression pattern in EAE

MiRNA has been found in exosomes isolated at all three time points during EAE course, early postimmunization (EAEp), at the peak of disease (EAEa) and at late time point of recovery (EAEl). We aimed to assess whether miRNA expression profile correlates with EAE development and could be associated with EAE induction, peak of clinical symptoms or disease resolution. We analyzed a set of samples from C57BL/6 mice induced with MOG. This model is more chronic compared to other EAE models, with significant similarities to progressive multiple sclerosis (7). In **Table 2A** we have shown miRNAs which were most upregulated at all three time points of EAE versus control mice. The largest group of significantly upregulated miRNAs (p<0.05 and fdr<0.05) was found at EAEp, the early time point of EAE (n=3). MiRNA control transcriptomic efficiency either by their up or down regulation, in **Table 2B** we have shown miRNAs which were most significantly downregulated in EAE versus control mice. Similarly to the analysis of the upregulated miRNAs, the largest number of downregulated miRNAs was found shortly after mice immunization (n=10), at EAEp time point. The data on up- and downregulated miRNAs demonstrate a dynamic sequential change in circulating exosomal miRNAs expression during EAE development with emphasis on the early stage of EAE. The pattern of expression of miRNAs during EAE suggests their active role in epigenetic regulation of cell transcription program contributing to induction of autoimmune process.

**Table 2A:** A List of the most significant upregulated miRNAs at different time points of EAE in C57BL/6 mice versus control mice.

EAEp vs control				EAEa vs control				EAEl vs control			
miRNA	P-value	logFC	FDR	miRNA	P-value	logFC	FDR	miRNA	P-value	logFC	FDR
mmu-miR-191-5p	0,0011	0,63	0,014	mmu-miR-28c	0,0516	1,35	0,0740	mmu-miR-7b-5p	0,00025	9,23	0,021
mmu-miR-451a	0,0055	0,81	0,042	mmu-miR-450a-5p	0,0811	1,27	0,1534	mmu-miR-7a-5p	0,0005	2,74	0,021
mmu-miR-2137	0,0077	1,40	0,05	mmu-miR-223-3p	0,0963	3,12	0,1672	mmu-miR-378d	0,0079	0,15	0,132
mmu-miR-339-3p	0,0121	0,87	0,073	mmu-miR-3068-5p	0,1312	0,93	0,2103	mmu-miR-5119	0,0157	1,34	0,243
mmu-miR-1897-5p	0,0207	0,32	0,084	mmu-miR-690	0,1706	0,79	0,2719	mmu-miR-378c	0,0159	0,97	0,252

**Table 2B:** A list of the most significant downregulated miRNAs at different time points of EAE in C57BL/6 mice versus control mice.

EAEp vs control				EAEa vs control				EAEl vs control			
miRNA	P-value	logFC	FDR	miRNA	P-value	logFC	FDR	miRNA	P-value	logFC	FDR
mmu-let-7f-5p	0,000065	-1,0	0,0055	mmu-miR-18a-5p	0,1111	-0.58	0,3399	mmu-miR-19a-3p	0,0317	-1,12	0,1469
mmu-miR-335-5p	0,0002	-1,4	0,0083	mmu-miR-1187	0,1399	-1,22	0,3399	mmu-miR-18a-5p	0,0555	-0,03	0,2005
mmu-let-7b-5p	0,00038	-0,93	0,0098	mmu-miR-8117	0,1593	-0,76	0,4737	mmu-miR-28a-5p	0,0556	-0,16	0,2027
mmu-miR-26a-5p	0,00047	-0,66	0,0098	mmu-miR-5130	0,2404	-1,56	0,5305	mmu-miR-183-5p	0,0556	-1,47	0,2027
mmu-miR-125a-5p	0,00066	-3,4	0,011	mmu-miR-19a-3p	0,2857	-2,01	0,5478	mmu-miR-124-3p	0,0720	-0,65	0,2920
mmu-let-7a-5p	0,0011	-1,2	0,014								
mmu-let-7c-5p	0,0014	-1,3	0,015								
mmu-miR-21a-5p	0,0039	-1,1	0,036								
mmu-miR-26b-5p	0,0046	-0,56	0,039								
mmu-miR-214-3p	0,0065	-2,4	0,045								

### Exosomal miRNA pattern expression in TBI models

MiRNA expression pattern in mechanical brain injury was analyzed in mice TBI model at two different time points post brain concussion, 3 (TBI3m) and 6 (TBI6m) months. On the whole miRNA expression at both these time points was significant diminished versus control mice (**Figure 2**). In **Table 3A** we have shown the most significantly upregulated miRNAs in TBI models at 3 and 6 months post brain injury and in **Table 3B** the most downregulated miRNAs versus control mice. The number of up- and downregulated miRNAs at both time points of TBI mice was larger in comparison to EAE mice. In TBI 3m group, 20 miRNAs were upregulated and 28 were downregulated. Accordingly, for the TBI 6m group 10 miRNAs were upregulated and 21 downregulated. Importantly, large number of the same up- and down regulated miRNAs occurred at both TBI time points indicating a similar trend in their expression regardless of time post brain injury. .

**Table 3A:** The list of the most upregulated miRNA at month 3 and 6 post TBI versus control mice.

TBI3m vs control				TBI6m vs control			
miRNA	P-value	logFC	FDR	miRNA	P-value	logFC	FDR
mmu-miR-451a	5,80E-07	0,765	0,000018	mmu-miR-142a-3p	0,0000059	1,7	0,00016
mmu-miR-486a-5p	0,0000087	1,519	0,00011	mmu-miR-451a	0,0000087	1,5	0,00018
mmu-miR-142a-3p	0,000011	1,972	0,00011	mmu-miR-10a-5p	0,000064	3,01	0,00083
mmu-miR-150-5p	0,000019	4,18	0,00018	mmu-miR-144-3p	0,00038	1,4	0,0024
mmu-miR-16-5p	0,00015	1,33	0,00093	mmu-miR-486a-5p	0,002	1,2	0,0082
mmu-miR-144-3p	0,00016	1,55	0,00097	mmu-miR-486b-5p	0,0022	1,21	0,0082
mmu-miR-106b-5p	0,00025	0,848	0,0012	mmu-miR-16-5p	0,0029	0,93	0,01
mmu-miR-20a-5p	0,00027	0,805	0,0012	mmu-miR-142a-5p	0,0063	2	0,019
mmu-miR-486b-5p	0,0006	1,527	0,0027	mmu-miR-7a-5p	0,0095	3,2	0,028
mmu-miR-10a-5p	0,0017	2,287	0,0062	mmu-miR-5119	0,011	2,2	0,029
mmu-miR-191-5p	0,003	0,226	0,0083				
mmu-miR-142a-5p	0,0039	1,802	0,012				
mmu-miR-484	0,0042	0,5468	0,011				
mmu-miR-5124a	0,0062	1,963	0,018				
mmu-miR-221-3p	0,012	0,7251	0,027				
mmu-miR-25-3p	0,015	2,437	0,034				
mmu-miR-98-5p	0,018	1,768	0,038				
mmu-miR-103-3p	0,022	0,4944	0,044				
mmu-miR-93-5p	0,022	0,359	0,044				
mmu-miR-186-5p	0,026	0,5721	0,048				

**Table 3B:** The list of the most downregulated miRNA at month 3 and 6 post TBI versus control mice.

TBI3m vs control				TBI6m vs control			
miRNA	P-value	logFC	FDR	miRNA	P-value	logFC	FDR
mmu-miR-184-3p	1,60E-07	-4,678	0,000012	mmu-miR-127-3p	1,10E-07	-7,7	0,0000092
mmu-miR-376c-3p	0,0000021	-6,58	0,00004	mmu-miR-143-3p	7,60E-07	-3,6	0,000031
mmu-miR-31-5p	0,0000061	-5,50	0,000094	mmu-miR-335-5p	0,000014	-3,5	0,00023
mmu-miR-125b-5p	0,000021	-1,353	0,00018	mmu-miR-6240	0,000071	-3,2	0,00083
mmu-let-7b-5p	0,000023	-1,64	0,0002	mmu-miR-99a-5p	0,000084	-4,8	0,00087
mmu-miR-127-3p	0,000038	-7,095	0,00029	mmu-miR-184-3p	0,000097	-4,7	0,00089
mmu-miR-27a-3p	0,00012	-1,572	0,00075	mmu-miR-27a-3p	0,00011	-1,1	0,00092
mmu-miR-143-3p	0,00014	-3,223	0,00093	mmu-let-7b-5p	0,00013	-1,5	0,00096
mmu-miR-100-5p	0,00019	-4,612	0,001	mmu-miR-125b-5p	0,00029	-3,2	0,002
mmu-miR-335-5p	0,0002	-2,575	0,0011	mmu-miR-320-3p	0,00046	-1,7	0,0027
mmu-miR-6240	0,00034	-2,416	0,0013	mmu-miR-199a-3p	0,00085	-3,2	0,0047
mmu-miR-145a-5p	0,00035	-1,09	0,0013	mmu-miR-199b-3p	0,00096	-3,1	0,0049
mmu-miR-141-3p	0,00045	-2,734	0,0016	mmu-miR-206-3p	0,0012	-4,7	0,0059
mmu-miR-27b-3p	0,00079	-1,572	0,0027	mmu-miR-190a-5p	0,0019	-2,8	0,0082
mmu-miR-206-3p	0,00097	-5,242	0,003	mmu-miR-145a-5p	0,0021	-3,5	0,0082
mmu-miR-320-3p	0,001	-1,756	0,0045	mmu-miR-141-3p	0,0022	-3,1	0,0082
mmu-let-7c-5p	0,0015	-1,264	0,006	mmu-miR-26a-5p	0,0036	-0,78	0,012
mmu-miR-190a-5p	0,0036	-3,111	0,012	mmu-let-7c-5p	0,0047	-1,1	0,015
mmu-miR-23a-3p	0,007	-1,013	0,019	mmu-let-7i-5p	0,006	-0,61	0,019
mmu-let-7j	0,0072	-2,61	0,019	mmu-miR-181a-5p	0,01	-1,9	0,029
mmu-miR-26a-5p	0,0076	-0,228	0,018	mmu-miR-376c-3p	0,012	-3,1	0,032
mmu-miR-99a-5p	0,0076	-2,529	0,02				
mmu-miR-181a-5p	0,0094	-2,547	0,023				
mmu-miR-199b-3p	0,016	-2,386	0,036				
mmu-miR-126a-3p	0,017	-0,776	0,037				
mmu-let-7a-5p	0,021	-0,8344	0,043				
mmu-miR-199a-3p	0,022	-2,416	0,044				

### Significant difference in exosomal miRNA expression pattern between EAE and TBI models

**Figure 3:**
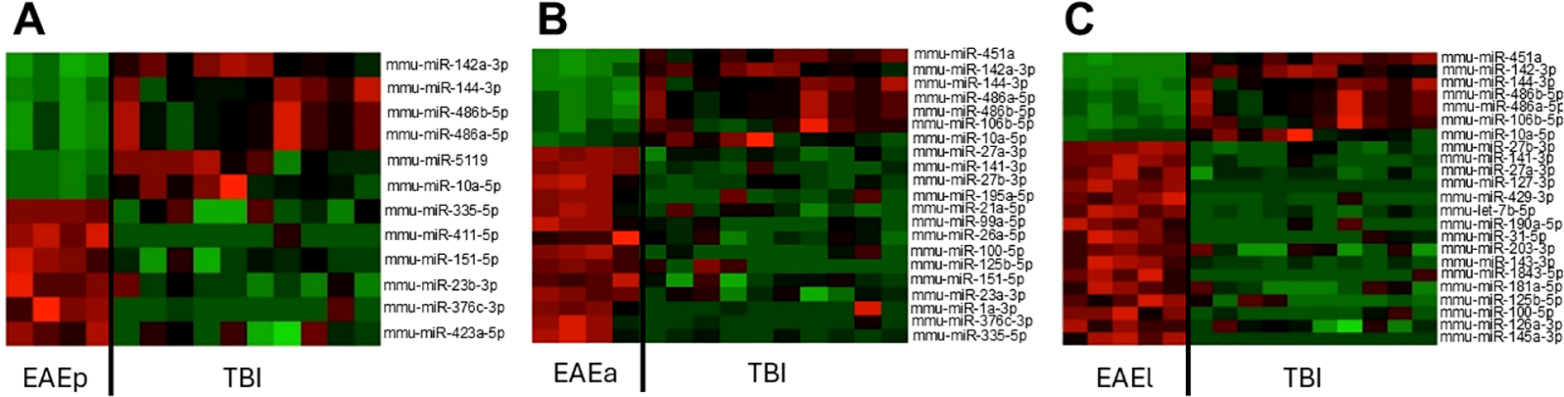
Heatmaps demonstrating differential expression of miRNAs at three EAE time points in C57BL/6 mice versus post TBI 3 months. **(A)** EAEp v TBI 3m, **(B)** EAEa v TBI 3m, **(C)** EAEl v TBI 3m.

Expression pattern of miRNA from EAE mice was significantly different from TBI models. On the whole, EAE mice at all three time points showed higher expression of circulating exosomal miRNA than TBI mice (**Figure 3**). The most upregulated miRNAs at each time point of EAE course, EAEp, EAEa and EAEl versus TBI 3m mice is shown in **Table 4A**. At EAEp only 2 miRNAs were upregulated versus TBI 3m, whereas at EAEa and EAEl 14 and 15 miRNAs showed upregulation, respectively. Five miRNAs, -27a-3p, -141-3p, -27b-3p, 100-5p and 125-5p were upregulated at the peak of EAE disease (EAEa) and at the recovery time point (EAEl). Significant lower number of miRNAs were downregulated in EAE versus TBI 3m (**Table 4B****).** Within the downregulated miRNA in EAE versus TBI 3m there was an interesting overlap of five species of miRNA, -142a-3p, -144-3-, -486a-5p, -486b-5p, -10a-5p which were downregulated at all three time points of EAE. These data may indicate relatively less selective miRNA downregulation in EAE vs TBI.

**Table 4A:** miRNA with highest upregulation at three time points of EAE course EAEp, EAEa and EAEl, versus TBI mice 3 months post-concussion.

EAEp vs TBI3m				EAEa vs TBI3m				EAEl vs TBI3m			
miRNA	P-value	logFC	FDR	miRNA	P-value	logFC	FDR	miRNA	P-value	logFC	FDR
mmu-miR-335-5p	0,0002062	1,31	0,0231036	mmu-miR-27a-3p	0,000000002	2,78	9,4937E-06	mmu-miR-27b-3p	0,00000002	2,90	0,00000093
mmu-miR-411-5p	0,0006364	4,23	0,0356404	mmu-miR-141-3p	0,000000013	3,05	2,522E-05	mmu-miR-141-3p	0,00000091	3,09	0,0012163
mmu-miR-151-5p	0,0019048	1,48	0,0640015	mmu-miR-27b-3p	0,000000046	2,27	5,5836E-05	mmu-miR-27a-3p	0,00015222	1,41	0,00759187
mmu-miR-23b-3p	0,0035266	1,58	0,0987464	mmu-miR-195a-5p	0,000001218	1,06	0,00073746	mmu-miR-127-3p	0,00020715	6,62	0,00918349
mmu-miR-376c-3p	0,0061374	3,68	0,1586292	mmu-miR-21a-5p	0,000006033	2,64	0,00273781	mmu-miR-429-3p	0,00028692	3,84	0,01040736
mmu-miR-423-5p	0,0063510	0,69	0,1524239	mmu-miR-99a-5p	0,000008160	0,86	0,00329146	mmu-let-7b-5p	0,00029842	1,59	0,00992254
				mmu-miR-26a-5p	0,00010987	2,97	0,00398835	mmu-miR-190a-5p	0,00032416	2,73	0,00994935
				mmu-miR-100-5p	0,00017784	1,88	0,0058687	mmu-miR-31-5p	0,00094328	2,76	0,02688354
				mmu-miR-125b-5p	0,00023357	1,10	0,0060562	mmu-miR-203-3p	0,00111977	1,33	0,02978583
				mmu-miR-151-5p	0,00031043	0,81	0,00751234	mmu-miR-143-3p	0,0017076	2,91	0,0425832
				mmu-miR-23a-3p	0,00036631	2,28	0,00831062	mmu-miR-1843b-5p	0,00182348	3,90	0,04279803
				mmu-miR-1a-3p	0,00038446	4,23	0,00820925	mmu-miR-181a-5p	0,00200131	1,71	0,04436238
				mmu-miR-376c-3p	0,00129508	3,12	0,02611754	mmu-miR-125b-5p	0,00210288	2,23	0,04416043
				mmu-miR-335-5p	0,00265711	2,78	0,05076473	mmu-miR-100-5p	0,00213851	2,98	0,04266328
								mmu-miR-126a-3p	0,00237544	0,69	0,04513335
								mmu-miR-145a-3p	0,00333745	1,21	0,06052927

**Table 4B:** miRNA with highest downregulation at three time point of EAE course EAEp, EAEa and EAEl, in versus TBI mice 3 months post-concussion.

EAEp vs TBI3m				EAEa vs TBI3m				EAEl vs TBI3m			
miRNA	P-value	logFC	FDR	miRNA	P-value	logFC	FDR	miRNA	P-value	logFC	FDR
mmu-miR-142a-3p	0,000012	-1,97	0,0040998	mmu-miR-451a	0,0000001	-2,02	0,00013559	mmu-miR-451a	0,00000011	-2,12	0,00022292
mmu-miR-144-3p	0,000162	-1,29	0,0272308	mmu-miR-142a-3p	0,0000003	-1,92	0,00028796	mmu-miR-142a-3p	0,00000104	-1,91	0,00104541
mmu-miR-486b-5p	0,000277	-1,41	0,0233207	mmu-miR-144-3p	0,0000021	-2,04	0,00113821	mmu-miR-144-3p	0,00000291	-2,02	0,00232918
mmu-miR-486a-5p	0,000294	-1,40	0,0197606	mmu-miR-486a-5p	0,0002155	-2,27	0,00651918	mmu-miR-486b-5p	0,00000909	-1,66	0,00604614
mmu-miR-5119	0,000896	-2,71	0,0430081	mmu-miR-486b-5p	0,0002247	-2,27	0,00627509	mmu-miR-486a-5p	0,00000943	-1,63	0,00537997
mmu-miR-10a-5p	0,001302	-3,64	0,0547216	mmu-miR-106b-5p	0,0027373	-0,95	0,04968340	mmu-miR-106b-5p	0,00022984	-1,36	0,00917065
				mmu-miR-10a-5p	0,0057449	-1,59	0,09067028	mmu-miR-10a-5p	0,00418718	-2,05	0,06961183

### Identification of the spectrum of transcript targets for circulating exosomal miRNAs

**Figure 4:**
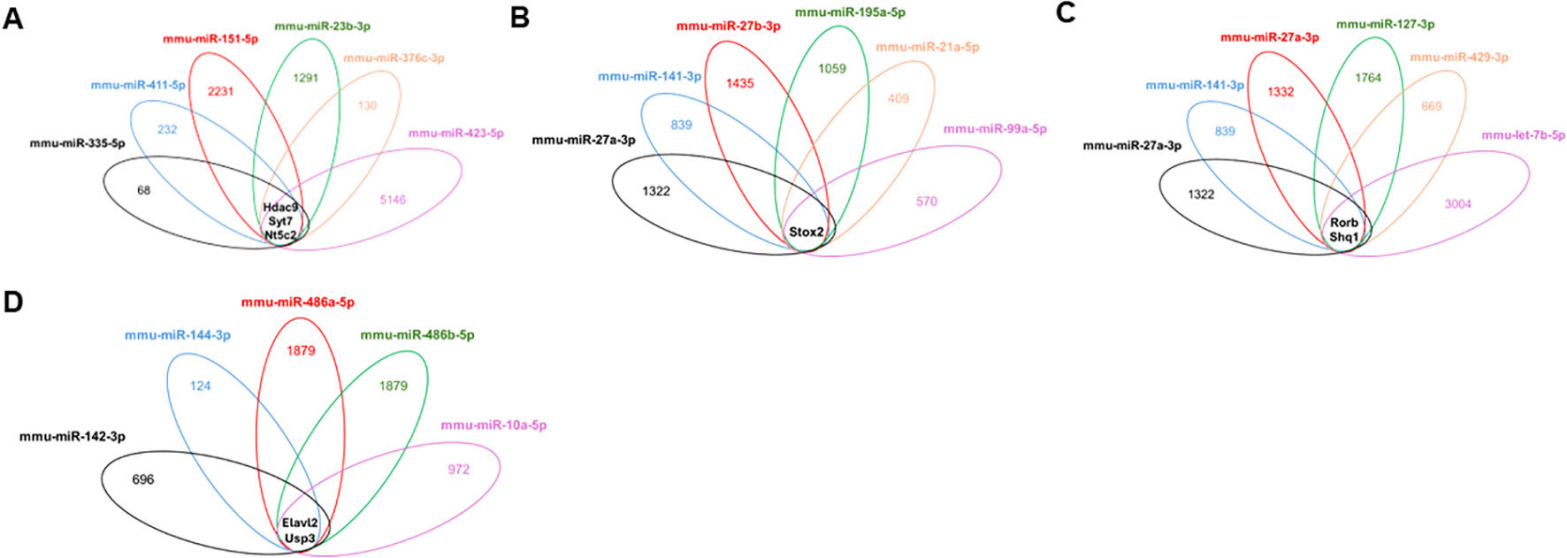
Protein transcripts controlled by the top differentially expressed miRNA in EAE versus TBI models. Each ellipse represents a number of transcripts regulated by each miRNA. The names of the transcripts shared by all miRNA are listed at the merging point of the ellipses. The figures represent top miRNA significantly upregulated in **(A)** EAEp v TBI, **(B)** EAEa v TBI, **(C)** EAEl v TBI. **(D)** Similar analysis of gene transcripts regulated by five top miRNA downregulated in either EAEp v TBI, EAEa v TBI and EAEl v TBI.

In an attempt to characterize the potential function of the differentially expressed exosomal miRNAs in EAE versus TBI models, we have analyzed the impact of these miRNAs on protein-coding transcripts. Analysis of the function of protein transcripts controlled by miRNAs differentially expressed in EAE and TBI models showed that the large class of encoded proteins belonged to transcriptional and DNA-interacting factors (**Figure 4**). Interestingly, several of identified protein genes have been associated with immune and inflammatory processes. Three protein transcripts, Hdac9, Syt7 and NT5c2 were identified in the early phase of EAE. All of them are linked with epigenetic switch controlling T cell fate and cytokine production. In recovery of EAE a RoRb, a member of ROR nuclear receptor family involved in Th17 cell differentiation was inhibited. Thus, the bioinformatic analyses of the putative gene targets of the exosomal miRNAs differentially expressed during EAE and TBI mice suggested their involvement in the CNS inflammatory processes both in EAE and TBI.

## Discussion

In this report, we have identified that circulating exosomal miRNA profiles showed a distinct pattern of expression between EAE and TBI, two models of different type of CNS pathologies, autoimmune versus mechanical injury. To provide an unbiased profile assessment, we started with a total exosomal RNA analysis using NGS. Thus, we obtained a complete transcriptome profile of circulating exosomes RNA in EAE and TBI models. We identified 10 different classes of exosomal RNA: miRNA, tRNA, piRNA, rRNA, lncRNA, mRNA, snRNA, yRNA, scRNA, other RNA. In the subsequent deep sequencing analysis and profiling of miRNA expression, we have found a number of miRNA which showed significant differential expression in EAE and TBI models.

Exosomes small membrane vesicles of endocytic origin are released from all types of cells. Importantly exosome content is not random but depends upon the parent cell status and their involvement in pathologic processes (6). They are known to contain a number of compounds, including proteins, lipids, mRNA, and miRNA, as well as double-stranded DNA. Therefore, analysis of circulating exosomes has been referred to as “liquid biopsy” to reflect the potential for insight into the structure and function of the donor cells without the need for their direct sampling (15). These results indicate that circulating exosomes and their content reflects the pathologic processes within CNS. The presence of CNS related molecules in circulating exosomes have already been reported (16), including our own study on myelin proteins in multiple sclerosis (17). MiRNAs are small, non-coding RNA molecules (about 20–24 nucleotides long) that play a key regulatory role in gene expression. They function primarily at the post-transcriptional level to fine-tune protein production within the cell (3). Among many activities, they control inflammation and activate or prevent overactive immune reactions (18). Interestingly, in both experimental models the number of miRNAs in circulating exosomes were lower than in control mice. In addition, more of miRNAs in EAE and TBI mice were downregulated than upregulated versus control animals. These data may suggest that the regulatory role of miRNA in transcriptional cell machinery in both EAE and TBI is decreased allowing translation of several proteins relevant to pathologic process.

Results of exosomal miRNAs expression pattern during EAE suggest their role already in early EAE events. However, subsequent dynamic change in miRNA expression over EAE course suggests also their involvement in more general reprograming of immune cells in autoimmune processes of EAE. The differentially expressed miRNA varied between the sequential three time points during EAE development: shortly after immunization, peak of disease and recovery indicating their specific contribution to all these stages of autoimmune disorder. These findings correspond to other recent publication on extracellular vesicles and their miRNA cargo in presymptomatic EAE mice (19). Considering the diversity of investigated models and clinical setups, it should be emphasized that many of differentially expressed miRNA found in our study corroborated with earlier reports on the up- and downregulated miRNA in EAE and multiple sclerosis e.g. miRNA 191-5p upregulated in MS (20), miRNA 7f-5p involved in regulation of Th17 pathway (21), miRNA 7b-5p with immune regulatory potential and interacting with EBV miRNAs (22), miRNA 26a-5p engaged in glutamate transporter expression (23), miRNA 125a-5p, miRNA 7a-5p, miRNA 26b-5p involved in oligodendrocyte progenitors differentiation (24). Consistency of these data may suggest the existence of unique pattern of miRNA environment in development of autoimmune demyelination.

The role of miRNA have also been already studied in TBI, both in clinical setups and experimental models. Substantial body of evidence demonstrated that miRNA might play a role in pathology of TBI including initial damage as well as in chronic secondary brain injury (25). MiRNA related to exosomes have directly been implicated in traumatic brain injury (26). We confirmed the presence of miRNA in exosomes from TBI mice. Interestingly, we have found that the pattern of miRNA up- and downregulated at 3 months post TBI showed extensive overlap with miRNA differentially expressed at 6 months post TBI suggesting long term contribution of miRNA-dependent regulatory mechanism in TBI. It should be admitted that we compare miRNA expression pattern in EAE with TBI with several weeks delay after brain injury. However, chronic traumatic encephalopathy represent an essential component of TBI, which might provide more data on the long-term outcome of mechanical brain injury.

With regard to differences in miRNA expression pattern between EAE and TBI models the list of the differentially upregulated miRNAs in EAE is quite heterogeneous. At the early stage post immunization (EAEp) two miRNAs were significantly upregulated, at the peak of disease (EAEa) and during recovery (EAEl) the number of significantly upregulated miRNAs was greater. Interestingly, the five most upregulated miRNA at the peak of disease, miRNA-27a-3p, miRNA 141-3p, miRNA 27b-5p, miRNA-100-5p and miRNA-125-5p showed also higher upregulation during recovery. In agreement with their expression in the recovery phase many of these miRNAs demonstrate immunoregulatory profile. MiRNA-27-3p is involved in targeting regulatory cytokines, IL-10 and TGF-b (27) and interacts with multiple intracellular signaling proteins involved in immune responses, including factors in NF-κB and MAPK pathways, influencing T-cell differentiation impacting TGF-β/SMAD signaling, which is important for Treg (regulatory T cell) development via FOXP3 expression and dysregulating TGF-β signaling inhibiting Th17 and Th1 differentiation (4), a key axis in MS immunopathology. MiR-141-3p reduces IL-1β, TNF-α, and IL-6 levels via targeting HMGB1 motifs in models relevant for CNS inflammation (28). The upregulation of these two immunoregulatory miRNAs already at the peak of the disease indicate early initiation of immunoregulation of EAE. The analysis of downregulated miRNA differentially expressed in EAE versus TBI showed interesting pattern of overlap of miRNA-142a-3p, miRNA144-3p, miRNA486a-5p, miRNA486b-5p and miRNA-10a-5p between all three EAE time points. Functional significance of these finding is supported with data on miRNA-142-3p which is highly expressed in T cells, macrophages, and dendritic cells, participating in antigen processing and presentation. Inhibition of miR-142-3p can promote anti-apoptotic and suppressive functions in Tregs by mechanisms involving chromatin modifications (29). Diminish expression of miRNA-144-3p was correlated with inhibition of pro-inflammatory effects in macrophages and decreased secretion of pro-inflammatory cytokines such as IL-1β, IL-6, TNF-α, CXCL2, and CCL2 (30). MiRNA-486a-5p and -486b-5p control immune cell activation and survival by regulating AKT signaling, influencing immune cell proliferation, apoptosis, and activation thresholds (31). Thus, the differential expression pattern of miRNA in EAE versus TBI correlates with their involvement in activation or inhibition of immune response.

The importance of miRNA-dependent regulation of EAE and TBI is supported with the results of bioinformatic analysis of protein coding genes controlled by differentially expressed miRNA. To strengthen this conclusions we presented the protein coding genes which were the target for all top differentially expressed miRNAs from each experimental groups. In the upregulated miRNAs at EAE early postimmunization time point (EAEp) three protein transcripts were identify, Hdac9, Syt7, Nt5c2. Hdac9 serves as an epigenetic switch controlling T cell fate and cytokine production (e.g., IL−4, CTLA−4) through histone deacetylation. Upregulation of Hdac9 resulted from downregulation of corresponding miRNAs in EAE diminish acetylation of transcriptional factors Foxp3 and STAT5 that decrease Treg stability and suppressive capacity (32), which can promote autoimmune inflammation. Interestingly Hdac has already been implicated in mechanisms of TBI through inhibition of neurotrophic factors expression and promotion of neuronal rewiring and functional recovery following TBI (33). Monitoring of relevant deacetylases with PET/CT studies in TBI rat model of diffuse, non-penetrating brain injury showed diminished Hdac activity in many brain regions (34). Syt7 is a Ca²^+^−sensing protein critical for synaptic vesicle replenishment and neurotransmitter release. MS−related study found that Syt7 expression and distribution are altered in gray matter lesions of MS patients, likely driven by upregulated miRNAs that target Syt7 mRNA (35). Transcriptomic analyses in trauma-associated neurodegeneration implicated synaptotagmins including SYT7, that were downregulated in a synaptic dysfunction module shared between chronic traumatic encephalopathy and Alzheimer’s disease (36). In addition, Syt7 controls multiple modes of synaptic vesicle exocytosis and plasticity which might be relevant to the source of miRNA from our study, extracellular circulating exosomes (37). NT5C2 encodes a cytosolic 5′−nucleotidase involved in nucleotide metabolism. Its relevance to MS immunity is primarily from its role in cladribine metabolism, modulating how immune cells process the drug and therefore influencing immune cell depletion in MS treatment (38). The only protein transcripts targeted by upregulated miRNAs from the peak of EAE disease (EAEa) was Stox2. It encodes a winged−helix transcription factor with roles in cell differentiation and development and can act as a co−factor in TGF−β signaling, which is a pathway with brofad roles in immunity and inflammation (39). At EAE recovery time point (EAEl) two protein transcripts were identified as target for upregulated miRNAs, Rorb and Shq1. Of particular interest is RORB a member of ROR nuclear receptor family (α, β, γ). Of these, RORC (encoding RORγt) is a critical transcription factor for Th17 cell differentiation and strongly linked to autoimmune diseases including MS (40). Shq1 encodes a protein involved in the biogenesis of H/ACA small nucleolar ribonucleoprotein particles (snoRNPs) and ribosomal RNA processing with no direct link with immunity (41).

The differentially downregulated miRNAs from EAE versus TBI mice interact with two protein transcripts, Elavl2 and Usp3. The first one is a neuronal RNA−binding protein expressed primarily in neurons in the brain. A recent immunology study (42) found that Elav2 controlled neuronal dsRNAs can activate pattern−recognition receptors (PRRs) such as MDA5, PKR, and TLR3, which are innate immune sensors triggering neuroinflammation. These results suggest that enhancement of neuronal regulatory process may contribute to EAE pathomechanisms. Expression of Elavl2 transcript has also been altered in TBI mice model and downregulated in neuronal stress (43). Interestingly, its expression was also link with Parkinson disorder (44). USP3 functions as a deubiquitinating enzyme regulating innate immune signaling (45). USP3 is a novel chromatin modifier that tightly regulates the DNA damage response. It acts as a key regulator of inflammatory vesicles and sustains the normal operation of the innate immune system (46). Its downregulation may indicate insufficient genomic integrity in TBI. USP3 also participates in innate immune tolerance by modulating pro−inflammatory signaling. It can deubiquitinate MyD88, an adaptor protein in Toll−like receptor and IL−1 receptor signaling, thereby inhibiting NF−κB activation and inflammatory cytokine production (47). Downregulated expression of USP3 in TBI may lead to heightened inflammatory signaling and increased pro−inflammatory cytokine release underlying the role of neuroinflammation in TBI. USP3-related deubiquitinases (USP11, USP30) were actively studied in TBI, highlighting the relevance of deubiquitylation in mechanical injury response (48).

The limitation of our study is related to the assessment of miRNA in circulating exosomes with limited information on CNS-derived exosomes. Thus we primarily found correlation between miRNA expression in circulating exosomes in two models of CNS injury. However, we believe that peripheral control of epigenic processes mediated by miRNA may play a role in these two CNS disease. In particular, immunization for EAE induction does occur in peripheral immune system.

Our data on exosomal miRNA expression in EAE and TBI correspond to their role in regulation of several molecular processes including neuroinflammation, an universal pathologic condition induced under different harmful stimuli representing an unique potential of CNS environment. Neuroinflammation represents classical pattern of EAE pathology but also contribute to pathologic mechanisms of TBI. Exosomes were shown to be generated by many cell types endogenous to the CNS, microglia, astrocytes, oligodendrocytes, endothelial cells. It has already been demonstrated that in response to CNS traumatic injury, exosomes mediate neuroinflammatory processes and contribute to tissue damage and repair (49). Although many mechanisms of neuroinflammation, like microglia activation, secretion of inflammatory cytokines and chemokines, astrocyte involvement, stimulation of Toll like receptors are common to various CNS pathologies (2), there are still important differences which might allow for individual meaning of miRNA epigenetic and biomarker values as well as their potential therapeutic targets. Since the pool of protein coding genes showed significant similarities in different types of neuroinflammation, the role of regulatory mechanism mediated by miRNA might play particularly important role in the scope of this process. Therefore, the finding that in classical neuroinflammatory condition like EAE, the pattern of miRNA expression differs from TBI induced neuroinflammation might allow for distinction between immune and non-immune regulatory mechanisms of neuroinflammation and consequently contribute to better understanding of miRNAs epigenetic regulation in different types of neuroinflammation including their biomarker role.

## Data Availability

Publicly available datasets were analyzed in this study. This data can be found here: [https://wl.uwm.edu.pl/knn/fotogaleria/mirnadata].
